# Documentation of Measles Elimination in Iran: Evidences from 2012 to 2014 

**Published:** 2017-08-05

**Authors:** Manoochehr Karami, Seyed Mohsen Zahraei, Azam Sabouri, Rambod Soltanshahi, Azam Biderafsh, Naser Piri, Jong-koo LEE

**Affiliations:** ^1^ Social Determinants of Health Research Center, Hamadan University of Medical Sciences, Hamadan, Iran; ^2^ Department of Epidemiology, School of Public Health, Hamadan University of Medical Sciences, Hamadan, Iran; ^3^ Center for Communicable Diseases Control, Ministry of Health and Medical Education, Tehran, Iran; ^4^ Departments of Social Medicine, Qom University of Medical Sciences, Qom, Iran; ^5^ Departments of Family Medicine, Seoul National University, College of Medicine, Seoul, Republic of Korea

**Keywords:** Infectious Diseases, Measles, Elimination, Reproductive number, Iran

## Abstract

**Background:** Documentation of achieving the goal of measles elimination to justify to international organizations including the WHO is a priority for public health authorities. This study aimed to address the status of Iran in the achievement of the measles elimination goal from 2012-2014.

**Study Design:** A descriptive study.

**Methods:** Data on the measles outbreaks were extracted from the national notifiable measles surveillance system in Iran from 2012 to 2014. The required documents regarding the achievement of measles elimination, including Effective Reproduction Number (R) and the distribution of outbreak size, was addressed. The R was calculated using the proportion of imported cases as 1 − P, where P is equal to the proportion of cases that were imported. The distribution of the measles outbreaks size was described using descriptive statistics to show their magnitudes. The proportion of large outbreaks with more than 10 cases was considered as a proxy of the R value.

**Results:** The total number of measles cases was 232 cases (including 186 outbreak related cases) in 2012 and 142 cases in 2014, including108 outbreak related cases. The distribution of the measles outbreak size of occurred outbreaks from that period indicated that there were 37 outbreaks with three or more than three cases. The R value in 2012 was 0.87 and the corresponding value for 2014 was 0.76.

**Conclusions:** According to the magnitude of effective reproduction number and distribution of outbreaks' size, measles has been eliminated in Iran. However, it is necessary to consider the potential endemic activity of measles because of no authorized immigration.

## Introduction

Measles is an acute and contagious respiratory infection caused by the measles virus^1^.The Islamic Republic of Iran is a member of the Eastern Mediterranean Region (EMR) region. Measles has been throughout history a well-diagnosed infectious disease and an important public health problem in Iran^2^.

Target dates for measles elimination in three WHO regions—the Americas, Europe, and the EMR—were set for 2000, 2007 and 2010, respectively^3^. Elimination is defined as the interruption of endemic measles virus transmission for a period ≥12 months in the presence of high quality and enhanced surveillance.

One approach to monitoring measles elimination involves estimating the basic and effective reproduction number (*R*). The basic reproduction number, commonly denoted by R_0_ , is "a key parameter that characterizes the early epidemic spread in a fully susceptible population"^4^. It can be used to inform public health authorities about the level of risk posed by an infectious disease and the potential effects of intervention strategies. Practically, temporal variation in the transmission potential of infectious diseases is monitored via the effective reproduction number, R_t_, is defined as the average number of secondary cases per primary case within calendar time^5^. If R_t_<1, then the epidemic be considered to have declined, while R_t_>1 indicates widespread transmission. The generalized-growth method^6^, as an alternative approach, could be used to characterize and estimate R_t_.

The aim of this study was to survey the status of Iran in the achievement of measles elimination from 2012 to 2014.

## Methods

Data on measles elimination were extracted from the measles surveillance system in Iran from 2012-2014. Surveillance system of measles elimination in Iran is one of the most important tasks of the Center for Communicable Diseases Control. Briefly, suspected cases of measles i.e. fever and rash cases are notified using an enhanced surveillance system by surveillance system staff. Details are described elsewhere^7^. There are no available data on the source of cases and history of measles immunization in 2013. Accordingly, we reported some specific indices for 2012 and 2014.

In this study, the required documents/indices regarding the achievement of measles elimination such as the number of cases imported into the country from neighboring countries, outbreak size distribution, distribution of the number of generations, country of acquisition, the number of occurred outbreaks, and the date of measles symptom onset were sought. In the absence of the date of symptom onset, the date of diagnosis was used.

There are three methods to estimate the effective R.* R* is the average number of secondary cases that result from an infectious case in a particular population^8^. *R* depends on the level of susceptibility in the population in contrast to the basic reproduction number (*R*_0_), which is the average of the secondary cases arising from one infectious case in a totally susceptible population. When *R* equals one, it is a state of endemic equilibrium in which, on average, a case results due to secondary infection. If the magnitude of *R* is greater than one, it means the number of cases increases from one generation to the next, potentially resulting in an epidemic. Moreover, when *R* equals to less than one, the number of cases decreases with each generation; and, if this value is maintained, elimination is considered to have occurred^8^.

In the present study, R was estimated as 1−P, where P was equal to the proportion of cases that were imported, as determined from data on the place of acquisition. If public health authorities believe that the measles cases had a foreign connection, based on international travel in the period before rash onset^9^, then the ailment was considered imported. This formula assumes that all cases must be linked to an imported case and the depletion of the pool of susceptible individuals can be ignored^8^.

Besides the calculation of an effective reproduction number as a document of measles elimination, we have utilized the distribution of the measles outbreaks size to show the magnitude of the outbreaks. The proportion of large outbreaks with more than 10 cases indicates the endemic activity of measles and a proxy of the R value. During the elimination phase of measles, a measles outbreak is defined as "the occurrence of one laboratory-confirmed measles case"^10^. It should be noted that results are exclusively related to confirmed cases of measles. In this study, we have categorized the reported source of occurred outbreaks as sporadic, endemic, imported related, imported relatives and unknown. If there were no sources as an origin of measles outbreak, this outbreak and related cases considered as sporadic cases.

Data were analyzed with Stata software version 12 (StataCorp LP, USA).

## Results

Of 232 measles cases in 2012, 186 outbreak related cases were assigned to known sources. About 76% of outbreak related cases were assigned to specific source of measles outbreak in 2014. The distribution of the characteristics of measles cases are shown in Table 1. The number of cases with source of origin as sporadic, endemic, imported related cases, imported relative cases, and unknown were 0, 65 (34.9%), 24 (12.9% ), 40 (21.5%), 51 (27.4%) in 2012. The corresponding sources of occurred outbreaks in 2014 was 0, 12 (11.1%), 32 (29.6%), 54(50%), 9(8.3%) as well. The number of vaccinated cases, those below the age of vaccination, unknown and unvaccinated were 63 (27.2%), 50 (21.5%), 33 (14.2%), 86 (37.1%) and 16 (11.3%), 33 (23.2%), 42 (29.6%), 51 (35.9%) in 2012 and 2014, respectively. Most of measles cases including outbreak related and imported cases were Iranian. Most cases were unvaccinated and under the age of vaccination. In addition, source of most cases were endemic and imported related cases in 2012 and 2014 respectively.

**Table 1:** Baseline characteristics of outbreak-related measles cases in Iran from 2012 to 201

**Characteristics**	**2012**		**2013**		**2014**	
	**Number**	**Percent**	**Number**	**Percent**	**Number**	**Percent**
**Source of cases occurrence** ^a^						
Sporadic	0	0.0	No data	No data	0	0.0
Endemic	65	34.9	No data	No data	12	11.1
Epidemic	6	3.2	No data	No data	1	0.9
Imported related cases	24	12.9	No data	No data	32	29.6
Imported relative cases	40	21.5	No data	No data	54	50.0
Unknown	51	27.5	No data	No data	9	8.3
**History of measles immunization**						
vaccinated	63	27.2	No data	No data	16	11.3
Under the age of vaccination	50	21.5	No data	No data	33	23.2
Unknown	33	14.2	No data	No data	42	29.6
Unvaccinated	86	37.1	No data	No data	51	35.9
**Nationality**						
Iranian	213	91.8	227	98.3	91	64.1
Afghan	17	7.3	4	1.7	50	35.2
Pakistan	2	0.9	0	0.0	0	0.0
Iraqi	0	0.0	0	0.0	1	0.7
**Place of residence**						
Urban	91	39.2	148	64.1	88	62.0
Rural	134	57.8	83	35.9	34	23.9
Itinerancy	7	3.0	0	0.0	7	4.9
Nomadic	0	0.0	0	0.0	13	9.2

^a^Imported cases were not included.

**Figure 1:**
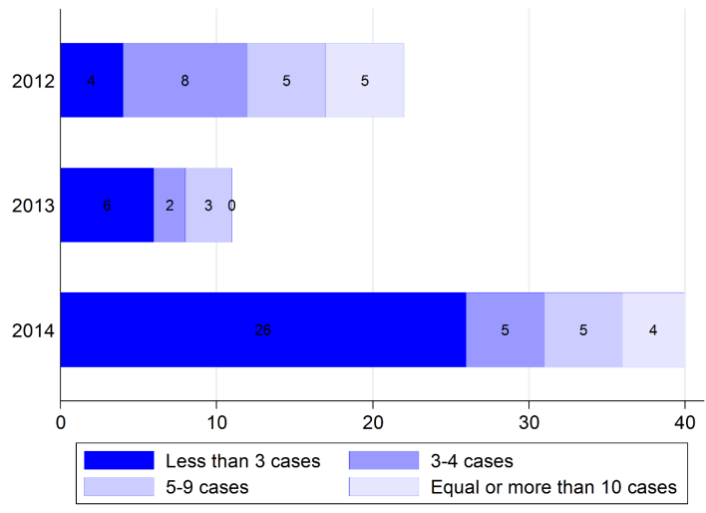
Distribution of the measles outbreak size in Iran from 2012 to 2014

The total number of outbreaks was more than 73 events in 2012 to 2014. Of them, 18 outbreaks with three or more than three cases occurred in 2012. The distribution of outbreaks, according to the outbreak size and year, are shown in Figure 1. Findings on the size of outbreaks during 2012 indicated that measles had been eliminated and about 22% of outbreaks had more than 10 cases. The size of the occurred outbreaks in 2014, too, supports view that measles elimination had been achieved.

The R value in 2012 with 24 imported cases was 0.87. This measure for 2014 among 142 cases with 34 imported cases was 0.76.

## Discussion

Our results indicate that most measles outbreaks were reported among the Iranian and Afghan nationals. Most cases were unvaccinated and under the age of vaccination, respectively. Additionally, most cases had source of endemic and imported related cases in 2012 and 2014, respectively. The findings on the size of the outbreaks during 2012 showed that measles was eliminated, and 22% of outbreaks had over 10 cases. In 2014, too, the size of occurred outbreaks supported the status of achieving measles elimination. Salimi et al.^2^ showed that majority of cases were male as 62.4% vs. 37.6% and only 20% of cases had history of vaccination. Moreover, 77% of the unvaccinated cases were less than two years of age. Six confirmed cases had come from Pakistan and Afghanistan^2^. Measles incidence had been reduced from 193/100,000 in 1981 to 6.8/100,000 in 2001^11^. Supplementary immunization activities have been done in many countries to increase the coverage of measles vaccination since 1994. However, immunization coverage of measles in some countries such as Afghanistan, Sudan, Somalia, Djibouti, and Pakistan was less than 60%^11^.

Measles immunization coverage in Iran has increased from 38% in 1980 to 99% in 2005 and percentage of measles immunization during more than 15 years was high, near to 99%^12^. Besides the role of sustained high coverage of measles vaccine, measles outbreaks with source-imported cases from neighbors are highlighted by published literature for Australia^13^. Same situation is dominant for Iran in case of travellers from neighbor countries with endemic activity of measles.

According to our results, the R value in 2012 with 24 imported cases and in 2014 with 34 imported cases were less than one. Accordingly, measles is being eliminated in Iran. R values based on the percentage of imported cases was in 2009–2012 about 66% (95% CI: 57%, 75%), 55% (95% CI: 43%, 67%), and 68% (95% CI: 62%, 75%)^14^.

Consider to the seasonality of measles, it is necessary to monitoring the measles activity using enhanced surveillance system during elimination phase. France during 2006 and 2007 is supposed to experience the elimination phase because of few reported cases. However, this country is affected by a large outbreak from 2008 to 2011 with more than 20, 000 measles cases^15^. Published literature in Iran was limited to the studies cited above, which aimed at demonstrating the epidemiological profile of measles diseases.

There is not available minimum required data on occurred measles outbreaks to appropriate linking imported cases or endemic cases. Moreover, there is no knowledge about genotypes of measles virus, which occurred during outbreaks in affected districts. The potential of missing some local outbreaks during elimination phase of measles by national notifiable diseases surveillance system is probable as well. Future studies should focus on other methods of R estimation to provide the best evidence on measles elimination in Iran.

## Conclusions

Measles had been eliminated from Iran. However, more up-to-date studies should be conducted on the status of measles activity in Iran.

## Acknowledgment

We would like to thank Dr. Mohammad Mehdi Gouya for his technical advise and support of this study.

## Conflict of interest statement

The authors declare that they have no competing interests to declare.

## Funding

The present study was approved and partially supported by the Center for Communicable Diseases Control, Iranian Ministry of Health and the Vice Chancellor of the Research and Technology of Hamadan University of Medical Sciences.

## Highlights

- Elimination of measles is achieved by Iran since 2012.
- Effective reproduction Number (R) is less than one in Iran in 2014.
- Besides Effective reproduction Number, size of occurred outbreaks in Iran supports that measles elimination had been achieved.
